# A Rare Case of Post-parathyroidectomy Calciphylaxis in a Young Patient With End-Stage Renal Disease: A Case Report and Literature Review

**DOI:** 10.7759/cureus.42937

**Published:** 2023-08-04

**Authors:** Valberto Sanha, Lennon Vidori, Beatriz C Pachi, Liana V Marchezi, Gisele Meinerz

**Affiliations:** 1 General Surgery, Federal University of Health Science of Porto Alegre, Porto Alegre, BRA; 2 Internal Medicine, Federal University of Health Science of Porto Alegre, Porto Alegre, BRA; 3 Nephrology, Federal University of Health Science of Porto Alegre, Porto Alegre, BRA

**Keywords:** end-stage renal disease, brown tumor, non-uremic calciphylaxis (nuc), parathyroidectomy, calciphylaxis

## Abstract

Calciphylaxis is a serious complication in chronic kidney disease (CKD) patients and requires a multidisciplinary approach. Dialysis patients are the most affected, especially those who are obese and diabetic, have liver diseases and autoimmune diseases, or are on systemic glucocorticoids. Parathyroidectomy (PTx) is curative for secondary hyperparathyroidism (SHPT) and calciphylaxis in most cases. Calciphylaxis after parathyroidectomy is a rare presentation. We present a case of a young patient who developed calciphylaxis after parathyroidectomy, an uncommon presentation.

## Introduction

Calcific uremic arteriolopathy or calciphylaxis is an uncommon cutaneous systemic disease that affects chronic kidney disease (CKD) patients. About 1%-4.5% of patients on dialysis are affected, especially those who are obese and diabetic, have liver diseases autoimmune diseases, or are on systemic glucocorticoids [1]. Usually, the patient initiates with a livedo reticularis-like skin lesion followed by violaceous and painful dark plaque, most frequently in the lower limbs, that progress to necrotic and ulcerative lesion [2]. In most cases, parathyroidectomy (PTx) is curative for secondary and tertiary hyperparathyroidism (HPT) and calciphylaxis. Calciphylaxis after parathyroidectomy is a rare presentation. Brown tumors (BT) occur due to the increased activity of the osteoclasts secondary to increased parathyroid hormone (PTH), which leads to local bone destruction, hemorrhage, and granulation tissue deposition containing giant cells in the regions of the rapid bone reabsorption [3]. Patients with secondary hyperparathyroidism (SHPT) can develop brown tumors. Here, we present a young female patient with an uncommon but severe complication of SHPT, and we review the literature on calciphylaxis post-PTx.

## Case presentation

**Figure 1:**
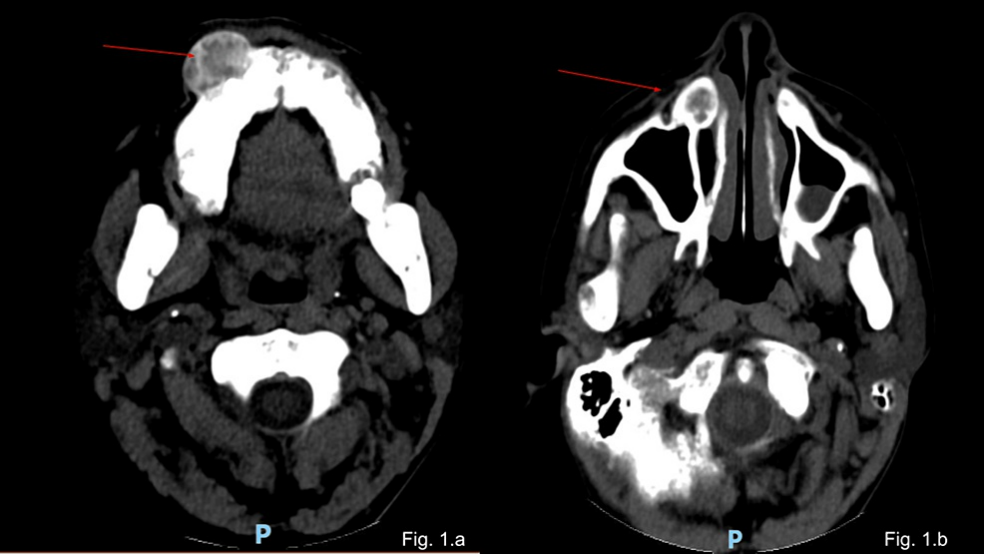
CT scan of the head Diffuse bone sclerosis related to renal osteodystrophy. Lytic bone lesion measuring approximately 2.2 × 1.7 cm located on the anterior surface of the right maxillary bone and the bulging of the adherent soft tissues suggestive of brown tumor

A 26-year-old female patient with a previous history of hypertension and end-stage renal disease (ESRD) due to chronic glomerulonephritis presented with painful maxillary bulging on her right cheek and facial edema noted weeks before coming to the emergency room. She rapidly progressed to renal replacement therapy after the initial diagnosis of her renal condition four years ago, and she has been on hemodialysis (HD) at our service since then. She had lean body composition, was not diabetic, did not take steroids, and did not have hepatitis or liver disease. She reported no dyspnea, dysphagia, or additional symptoms. Oral cavity examination showed bulging in the gingival region in the upper right jaw. Head CT scan on admission was consistent with a brown tumor (Figure 1a, 1b). Doppler ultrasound of the thyroid gland evidenced three nodules suggestive of parathyroid hyperplasia. There was a persistent elevation of PTH of >2000 pg/mL for more than a year, despite clinical treatment with calcimimetics. Due to the persistent elevation of PTH and the clinical repercussions of secondary hyperparathyroidism, total parathyroidectomy with forearm autotransplantation and partial right-side maxillectomy was performed. Preoperative intact PTH (iPTH) was >2000 pg/mL, intraoperative PTH was 882 pg/mL, and PTH was 43 pg/mL on the first postoperative day, 9.3 pg/mL on the third postoperative day, and 118 pg/mL four weeks later (normal PTH reference range: 10-52 pg/mL).

**Figure 2:**
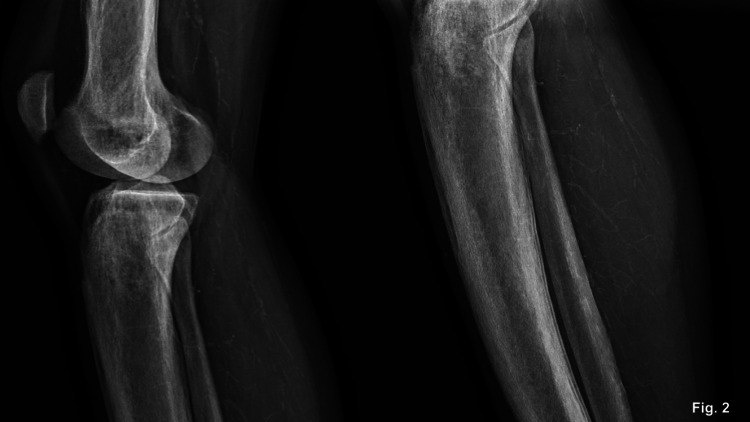
Limb X-rays were ordered to evaluate calcium deposition, which identified vascular calcification on lower extremity vessels

**Figure 3:**
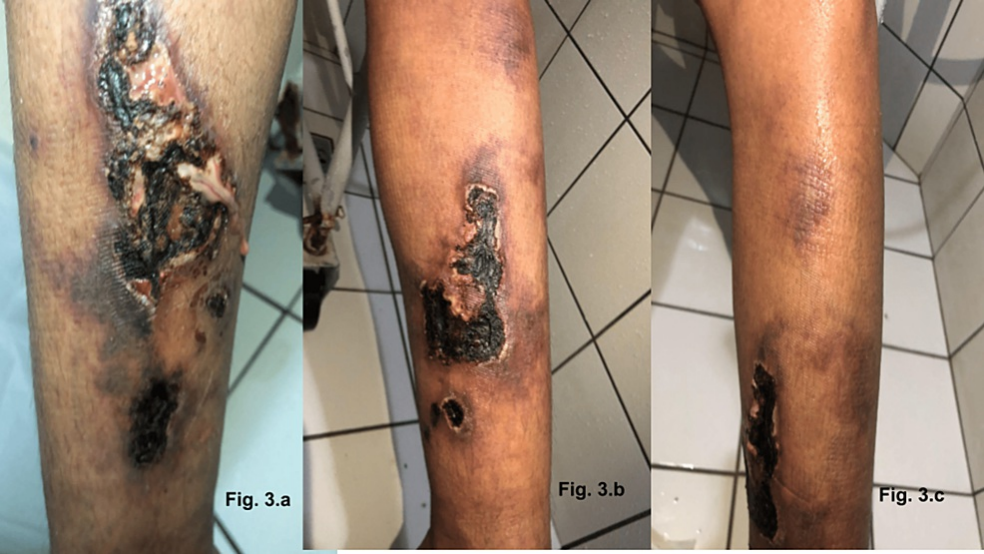
Necrotic ulcerated skin lesion with fluid secretion in the medial aspect of the right leg and in the contralateral leg

**Figure 4:**
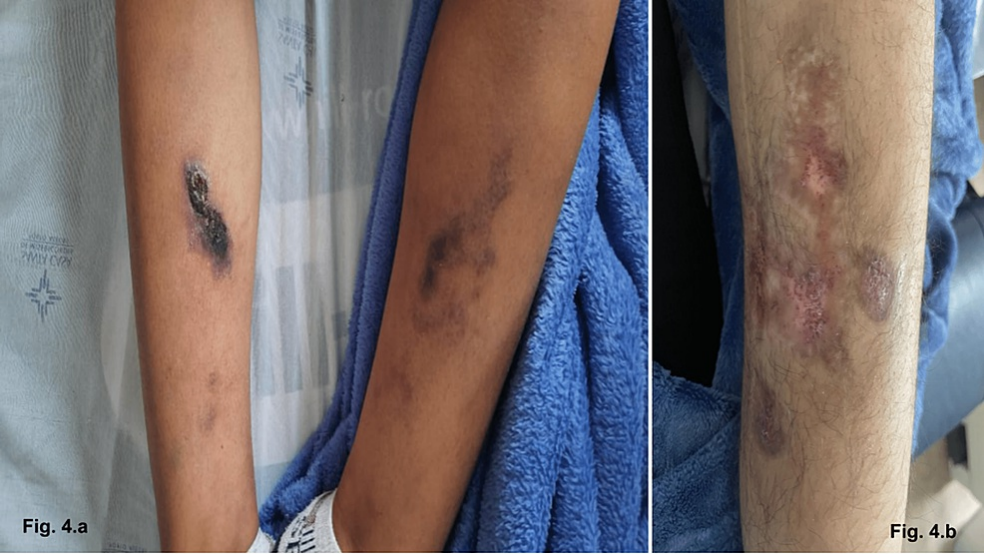
Significant improvement of the skin lesions progressing to a complete resolution of the lesions

The histopathological examination of the maxillary bone lesion was consistent with a brown tumor. She developed hungry bone syndrome after the procedure and required intravenous (IV) calcium for a few days and was discharged with oral calcium tablets. Three weeks after the surgical procedure, she noticed an itchy nonulcerated single dark spot in the medial aspect of the right leg and returned to the emergency room. The initial dermatologic evaluation described a single painful hyperchromic and purpuric lesion without skin ulcerations or fluid secretion in the medial aspect of the right leg. Due to the concern about calcium deposition, a skin biopsy and limb X-rays were ordered. The skin biopsy revealed the findings of stasis dermatitis but no foci of dermal dystrophic calcification on tissue obtained, and X-rays identified vascular calcification on lower extremity vessels (Figure 2). In the following weeks, the patient reported worsening of the pain and the enlargement of the previous dermatologic lesion, now ulcerated, and the appearance of a new lesion in the contralateral leg (Figure 3a-3c). The characteristic of the skin lesion and the clinical presentation strongly suggested calciphylaxis, and sodium thiosulphate was introduced. No forearm graft hyperplasia or ectopic residual gland was identified on further workup. The necrotic lesion continued to worsen rapidly, and a new ulcerated skin lesion appeared, and pain management and wound care were intensified. She later developed a secondary infection, which warranted IV antibiotics and wound debridement. After initial improvement, the patient was discharged and continued to be followed closely in the outpatient clinic and continued appropriate wound care and hemodialysis in our institution. Several weeks later, with appropriate management for calciphylaxis, the patient presented great improvement in the skin lesions, which progressed to complete healing (Figure 4a, 4b).

## Discussion

Calciphylaxis is a feared complication in ESRD patients on renal replacement therapy, and its management requires a multidisciplinary approach. It is very unlikely to happen after parathyroidectomy. There are limited cases described in the literature. Table 1 summarizes the reported cases to date [4-10]. Secondary hyperparathyroidism (SHPT) is a common complication in CKD patients on HD, and up to 90% of patients on HD will develop SHPT, and about 1.5% will develop brown tumors [11,12]. Histologically, BT can mimic giant cell tumors with a brown-colored lesion due to hemosiderin deposition. A brown tumor is more common in the mandible than in the maxilla and is more frequent in females around their 50s, especially postmenopausal females [11,13]. The presence of a brown tumor at a younger age is uncommon, and so is calciphylaxis after parathyroidectomy. Herein, we present a case of a young patient with a brown tumor who developed severe calciphylaxis after parathyroidectomy. These findings distinguish our case from the others.

**Table 1:** Table of the cases reported in the literature PTx, parathyroidectomy; PTH, parathyroid hormone; HPT, hyperparathyroidism; SHPT, secondary hyperparathyroidism; iPTH, intact parathyroid hormone

Author and year of publication	Patient's gender	Age	Hemodialysis	PTx indication	Pre-PTx level of PTH	Type of procedure	Post-PTx level of PTH	Distribution of the lesions	Occurrence time after PTx	Diagnostic methods	Post-biopsy complication
Poch et al., 1992 [4]	Male	62	Yes	Osteodystrophy and bone pain due to SHPT	1205 pg/mL	Subtotal PTx	213	Distal lower extremities	3 months	Skin biopsy	Not described
Oikawa et al., 2004 [5]	Male	32	Yes	SHPT	Unknown	Parathyroidectomy (not specified)	63 during the onset of symptoms	Lower abdominal wall and penile involvement	2 years	Skin biopsy	Not described
Bonilla et al., 2007 [6]	Female	59	Yes	Calciphylaxis	Unknown	Subtotal PTx	Unknown	Breast	Unknown	Skin biopsy	Total mastectomy for nonhealing wound after an open breast biopsy
Wahab et al., 2008 [7]	Male	33	Yes	SHPT	100.7 pg/mL (370 pmol/L)	Total with sternocleidomastoid implantation of the remaining gland	5.05	Distal lower extremities	7 weeks	Evidenced in skin biopsy	Not described
Katikaneni et al., 2013 [8]	Female	62	Yes	Calciphylaxis	362 pg/mL	Subtotal PTx	406	Breast, thigh, and lower abdominal wall	16 months	Skin biopsy	Not described
Bashir et al., 2016 [9]	Male	46	Yes	Pathological fracture due to SHPT	2000 pg/mL	Subtotal PTx	891	Penile	2 weeks	CT scan	Biopsy was avoided, good outcome; patient penile lesion healed
Karmegam and Shetty, 2017 [10]	Male	60	Yes	HPT and osteopenia	4191 pg/mL	Subtotal PTx	184	Lower extremity and buttocks	4 weeks	Skin biopsy	Lesion progressed; sepsis; despise
Our study, 2023	Female	26	Yes	Brown tumor due to SHPT	>2000 pg/mL	Total PTx + forearm autotransplantation	Intraoperative iPTH of 882 pg/mL and iPTH of 43 pg/mL on the first postoperative day	Distal lower extremities	3 weeks	Clinical diagnosis (negative early biopsy)	Wound infection and expansion of the lesion

Calciphylaxis can present with a variety of skin lesions. A definitive diagnosis can be established through a skin biopsy, which can demonstrate calcium deposition in the medial layers of small vessels and in the extravascular soft tissue, along with other specific characteristics. A negative skin biopsy does not necessarily exclude the diagnosis of calciphylaxis [14,15]. In Nigwekar et al.'s study [16], they described a retrospective study with 1030 patients on hemodialysis who later developed calciphylaxis. About 55% of the patients had positive skin biopsies, whereas 45% had a negative biopsy and were diagnosed clinically in that study. In most cases, clinical characteristics can be sufficient to establish the diagnosis, especially in patients with known risk factors. A skin biopsy should be reserved for cases of uncertain diagnosis as it imposes an additional risk of skin ulceration, propagation of the lesion, and superimposed infection. Moreover, the sensitivity of the procedure can be influenced by the time it is performed, the depth of tissue specimen and processing, and the experience of the surgeon or dermatologist; early biopsy may be associated with low sensitivity [14].

The patient's initial skin biopsy at arrival was negative for calciphylaxis. In the next few days, the clinical course helped us establish the diagnosis and manage the condition accordingly despite the negative skin biopsy. In our retrospective analysis of the case, we believe that the skin biopsy could have been delayed and ultimately not even performed since it did not help define the diagnosis, and conversely, it worsened the lesion and probably favored the skin infection. The fact that the lesions started after PTx certainly drove us off track, as it was unexpected. We highlight here the unusual presentation of an uncommon condition. Reviewing the reported cases of post-PTx calciphylaxis, most cases were diagnosed by skin biopsy, even ESRD patients with characteristic skin lesions. Bashir et al. described a case of post-PTx calciphylaxis where they avoided skin biopsy due to the fear of the expansion of the lesion, the overall outcome was favorable, and the lesion healed completely [9]. On the contrary, in some cases where a biopsy was performed, procedural complications were identified. Like Bonilla et al.'s study [6], an open breast biopsy was performed for the diagnosis and culminated with total mastectomy due to a nonhealing ulcerated breast wound that developed after the biopsy. These findings highlight the importance of carefully weighing the benefits and the downsides of skin biopsy by the dermatologist and close discussion with the patients.

A persistent elevation of PTH in secondary hyperparathyroidism with parathyroid bone diseases is an indication for the surgical removal of hyperplastic glands. Either subtotal PTx or total PTx with autotransplantation can be performed. Despite PTx, many patients might still develop recurrent hyperparathyroidism [17]. However, it is uncommon to develop calciphylaxis after parathyroidectomy. Our patient had a formal indication for parathyroidectomy, so the procedure was done. Unfortunately, she developed progressive skin lesions consistent with calciphylaxis weeks after parathyroidectomy, a rare complication.

In our literature review of the previous cases reported on calciphylaxis after parathyroidectomy, we found only a few cases. Table 1 is a summary of cases reported to date. The mean age in the reported cases is 50.5 years old, most of the patients were male, and all of them were ESRD patients and on dialysis therapy. Indication for PTx was in the majority due to persistent hyperparathyroidism. In two cases, Bonilla et al. [6] and Katikaneni et al. [8], calciphylaxis was the indication for PTx. These patients' initial skin lesions resolved after PTx and developed new proximal calciphylaxis; the breast was involved in both at some point after the surgery. Bonilla et al. found a residual gland that was responsible for the recurrence of HPT [6]. There was no description of the PTH level at the moment of the recurrence of the calciphylaxis. In the rest of the cases, no skin lesions were found prior to the procedure, and these patients later developed skin lesions. The underlying mechanism of calciphylaxis after parathyroidectomy is not fully understood yet. For some authors, a decrease in bone turnover due to an abrupt reduction in PTH after PTx results in increased serum calcium and phosphate concentration that would have been used for bone mineralization in a high bone turnover state. This increase in calcium phosphate causes vascular deposition and calciphylaxis seen after parathyroidectomy [10]. Very high levels of PTH (>1000 pg/mL) were reported in almost all cases of calciphylaxis post-parathyroidectomy (Table 1). Only one case had a pre-surgical PTH of 370 pg/mL, and two cases did not describe the PTH level prior to surgery. The higher the pre-PTx PTH, the more intensified the bone turnover rate. An abrupt decrease in the PTH level will cause more significant differences in the pre- and post-procedural levels of PTH. Thus, these patients will have an increased risk of calciphylaxis, as seen in the reported cases.

## Conclusions

Calciphylaxis after parathyroidectomy is a rare entity; however, clinicians need to be aware of it. This present case is very important because it highlights the two uncommon complications that can occur in ESRD patients. It also highlights the role of dermatologists in avoiding unnecessary biopsies when the clinical picture strongly suggests the diagnosis due to the risk of complication that biopsy renders plus the low sensitivity in some situations.
